# Intrahepatic eosinophilic proliferative phlebitis in Japanese black cattle indicate allergies involving mast cell tryptase-dependent activation

**DOI:** 10.3389/fvets.2022.972180

**Published:** 2022-12-20

**Authors:** Masaki Konnai, Kimimasa Takahashi, Yukino Machida, Masaki Michishita, Kozo Ohkusu-Tsukada

**Affiliations:** Department of Veterinary Pathology, Nippon Veterinary and Life-Science University (NVLU), Tokyo, Japan

**Keywords:** mast cell tryptase, interleukin-4, proteinase-activated receptor-2, food allergy, Japanese black cattle

## Abstract

Cow-specific feature hepatic lesion, termed as eosinophilic proliferative phlebitis (EPP), has been mainly detected in Japanese black cattle and identified histologically eosinophilic infiltration and endothelial hyperplasia in portal areas. We previously proposed EPP as a food allergy from the pathological characteristics and a significant increase of serum immunoglobulin E specific to curly dock (*Rumex crispus*) in allergens testing, however, first report had regarded EPP an atypical type of bovine fascioliasis. In EPP lesions, eosinophilic infiltration was observed to the hypertrophic endothelium and not to the intrahepatic bile duct, and that was related to eotaxin-1 expression. In EPP, the mast cells increased as well as in fascioliasis, and the mast cells producing tryptase without chymase increased with interleukin-4 production. In this context, hyperplasia of periendothelium expressing proteinase-activated receptor-2 (PAR-2) and not angiotensin II was observed. Contrastably, in fascioliasis, unique mast cells producing neither tryptase nor chymase infiltrated, and the periendothelium expressed neither PAR-2 nor angiotensin II. Interestingly, EPP had not occurred liver injury with raised hepatic enzymes like fascioliasis, and suggested to a correlation with severe serum hypo-vitamin A. Overall, this study suggests that EPP is an allergic disease by main difference between adaptive immunity to allergens and innate immunity to parasites.

## 1. Introduction

The occurrence of feature hepatic lesion is sometimes seen in Japanese black (JB) cattle, a breed of beef cattle in Japan. Usually, when such hepatic lesions are detected with a high incidence of 0.4–0.5% (no gender difference) in meat hygiene inspection in Japan, regardless of JB cattle being brought in slaughterhouses as healthy cattle, only the liver is discarded (1). The first case of this hepatic lesion was reported as an atypical form of bovine fascioliasis with no sign of interlobular cholangitis and termed as intrahepatic eosinophilic proliferative (pyle-)phlebitis (EPP) (2). In Japan, however, there is almost no grazing, so it is unlikely that bovine fascioliasis will epidemic. Furthermore, despite the worldwide prevalence of bovine fascioliasis, it remains unclear why EPP cases only occurs in Japan.

We previously proposed hypersensitivity to forage as the mechanism for EPP pathogenesis, which indicated a marked increase in the serum immunoglobulin E (IgE) specific to curly dock (*Rumex crispus*) among antigens screened for allergen profiling compared with healthy or fascioliasis cattle (3). Curly dock is extensively naturalized throughout the temperate climate regions and thrives in various habitats, including disturbed soil, waste areas, roadsides, shorelines, forest edges, fields/meadows, and even well-maintained farms, thereby increasing the likelihood of contamination of curly dock in forage harvested from farms (4). Recent reports suggested a correlation between an allergen of the curly dock and the pathogenesis of allergic dermatitis in horses (5) and allergic rhinitis in humans (6).

The purpose of this study is to clarify whether EPP cattle are allergic diseases or non-major type of fascioliasis. Because the mechanisms of eosinophilic infiltration have been distinguished from adaptive immunity through IgE-crosslinked mast cell (MC) activation to allergens and innate immunity through Group-2 innate lymphoid cells (ILC2) to a parasite response (7–9), we investigated MCs producing interleukin-4 (IL-4) and specific granules (chymase and/or tryptase), angiotensin II (Ang II) and/or protease activated receptor-2 (PAR-2)-expressing periendothelium fibroblasts, and eotaxine-1-producing cells (endothelium cells, fibroblasts, etc.) in relation with food allergy or bovine fascioliasis.

## 2. Materials and methods

### 2.1. Sample collection

We obtained liver samples of 10 JB cattle with EPP (age: 25–35 months; all female), six JB cattle with fascioliasis (age: 98–169 months; all female), and five healthy JB cattle (age: 27–33 months; all female) from the following Japanese meat inspection centers in Iwate, Kanagawa, and Yamagata Prefectures. Although each animal with EPP lesions was designated healthy in both a routine on-farm examination and a definitive checkup in slaughterhouses conducted by veterinarians, the detected morbid livers were disposed when they became a subject of administrative partial waste after dismantlement. The morbid liver samples were obtained after they were disposed as waste. Five healthy hepatic samples were purchased from the meat inspection centers in Kanagawa Prefecture (Yokohama area) in which the recommendations of VA-deficient feeding were not implemented. If flukes were observed upon the inspection of the liver, the samples were determined to be *Fasciola* samples. For confirmation, the presence of anti-*Fasciola hepatica* immunoglobulin G (IgG) was tested in all serum samples using a commercially available enzyme-linked immunosorbent assay kit (BIO K 211; Bio-X Diagnostics) according to the manufacturer's instructions. Positive results were obtained for all fascioliasis cases, whereas negative results were obtained for any EPP and healthy cases.

### 2.2. Immunohistochemistry

Two or three sections of every sample (thickness, 1 micron) were dewaxed and rehydrated. Sections were covered with proteinase K working solution and incubated for 15 min at 37°C in a humidified chamber to break protein cross-bonds of aldehyde with formalin-fixed. Endogenous peroxidase activity was blocked by immersion in 0.3% H_2_O_2_ in methyl alcohol for 15 min at room temperature (RT). After antigen retrieval, cooling at RT was performed for 20 min. Non-specific antigenic sites on slides were blocked *via* incubation in a 25% Block ACE (Bio-Rad) solution for 30 min at RT. Subsequently, incubation overnight at 4°C was performed with the primary antibodies (Abs). The primary Abs used were mouse monoclonal Abs against MC tryptase (Clone AA1, dilution 1:500, GenTex), IL-4 (Clone 1G2C5, dilution 1:200, Proteintech), MC Chymase (Clone B7, dilution 1:200, Merck), and rabbit polyclonal Abs against human Eotaxin (dilution 1:100, LSBio), Ang II (dilution 1:200, Arigo), PAR-2/F2RL1 (dilution 1:200, Aviva system biology). Subsequently, sections were incubated with biotinylated goat anti-mouse or anti-rabbit IgG Abs (DAKO). After this step, peroxidase-conjugated streptavidin (DAKO) was used for 30 min at RT. Finally, diaminobenzidine tetrahydrochloride chromogen (DAB) was added to make the reactions of each antigen evident, and the slides were counterstained with hematoxylin. These steps without the primary Abs were performed as negative control. The tissue images were captured using a microscope imaging software (cellSens, OLYMPUS). The number of cells per1 mm^2^ was calculated using total count numbers in 10 random fields of an image of 274 × 365 μm.

### 2.3. Serum analysis

Serum samples of Healthy (*n* = 5), EPP (*n* = 10), and Fascioliasis cattle (*n* = 6) were analyzed about vitamin A (VA) (retinol, IU/dl), aspartate aminotransferase (AST, IU/l), and γ-glutamyl transpeptidase (γ-GTP, IU/l) by LSI Medience Co., Ltd.

### 2.4. Statistical analysis

Data were statistically evaluated using analysis of variance (ANOVA) using Microsoft Excel (Ver.16.61.1). *P-*value < 0.05 was considered statistically significant.

## 3. Results

### 3.1. Characteristic hepatic lesions in EPP and fascioliasis

**Figure 1:**
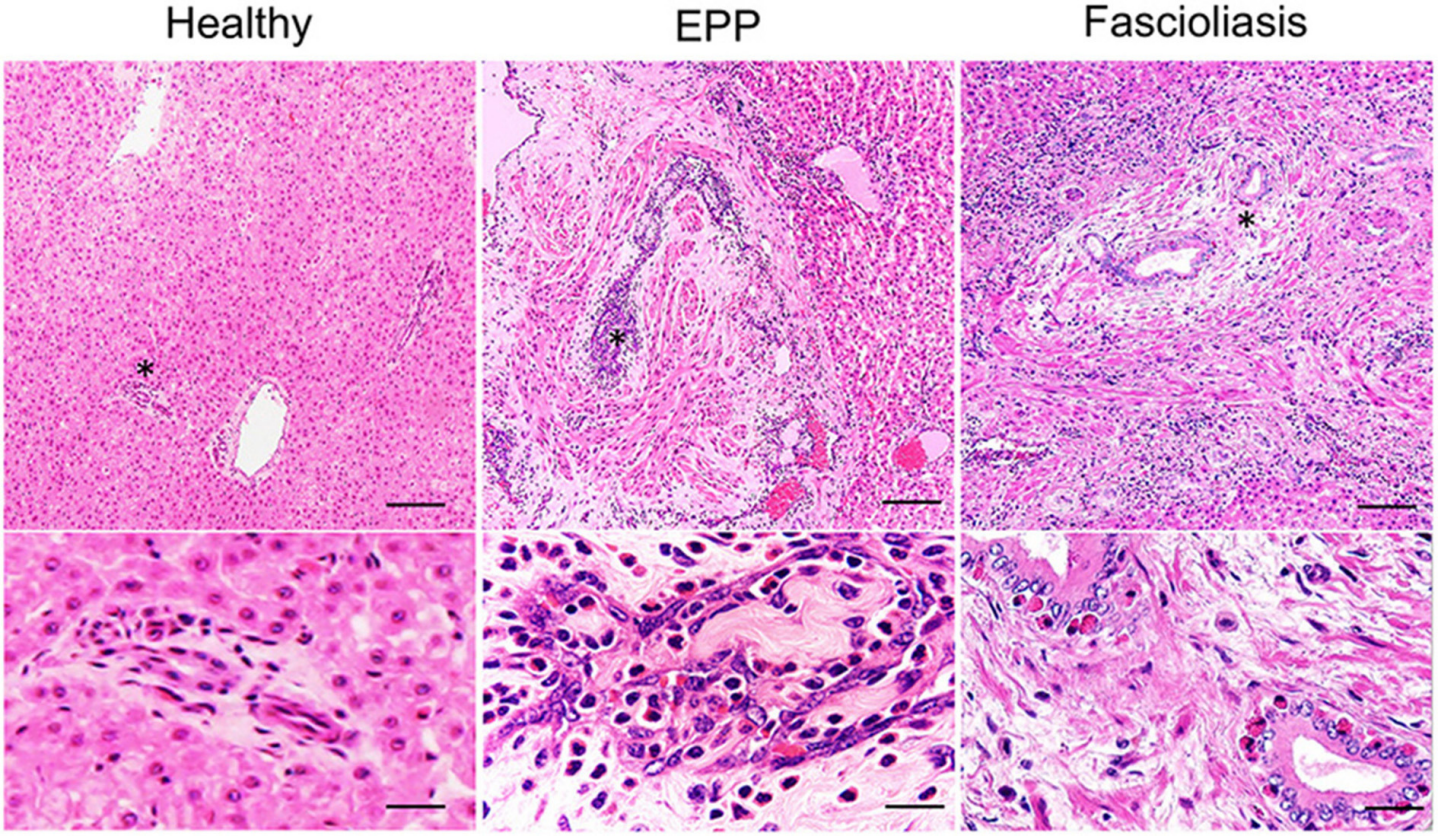
Hematoxylin–eosin (HE) staining images of the hepatic interlobular triad region in a healthy liver, an eosinophilic proliferative phlebitis (EPP) case, and a chronic fascioliasis case. Mild eosinophilic infiltration is observed in the hepatic interlobular triad area in healthy cattle (Healthy). In EPP cattle, portal vein branches show irregularly dilated lumens and aberrant intimal proliferation, with concomitant extensive endothelial hyperplasia, intimal fibrosis, vascular smooth muscle hyperplasia, and severe eosinophilic infiltration in the endothelium. In fascioliasis cattle (Fascioliasis), the biliary epitheliums show papillary and glandular hyperplasia, accompanied by fibrosis and accumulation on the biliary epithelium by moderate infiltration of mononuclear cells and eosinophil. High-magnification image is indicated at the bottom with an asterisk (*). Each scale bar indicates 50 μm in low-magnification images and 10 μm in high-magnification images.

In healthy cattle, even in livers that appeared normal, a small number of eosinophils and mononuclear cells were often detected in the portal areas histologically. In EPP cattle, the liver samples indicated gross lesions lobed-like with thickened and protruding vessels. Histologically, walls of the portal vein branches were unevenly thickened along with remarkable endothelial hyperplasia, intimal fibrosis, vascular smooth muscle hyperplasia, and severe eosinophilic infiltration in the endothelium (Figure 1). In addition, centrilobular veins, hepatic artery branches, and bile ducts were mostly unmodified in the presence of lesions in the portal vein branches. In contrast, liver samples in fascioliasis cattle were diagnosed by direct visualization of mature liver flukes (*Fasciola* spp., a crossbreed of *F. hepatica* and *F. gigantica*) in the larger bile ducts (Figure 1). Histologically, we observed papillary and glandular hyperplasia of the biliary epithelium along with moderate eosinophilic infiltration in the biliary epithelium, calcification, fibrosis, and accumulation of mononuclear cells (data not shown). These findings suggest that EPP and fascioliasis have completely different lesion formation processes in interlobular veins and interlobular bile ducts.

### 3.2. Number of total and IL-4–producing MCs

**Figure 2:**
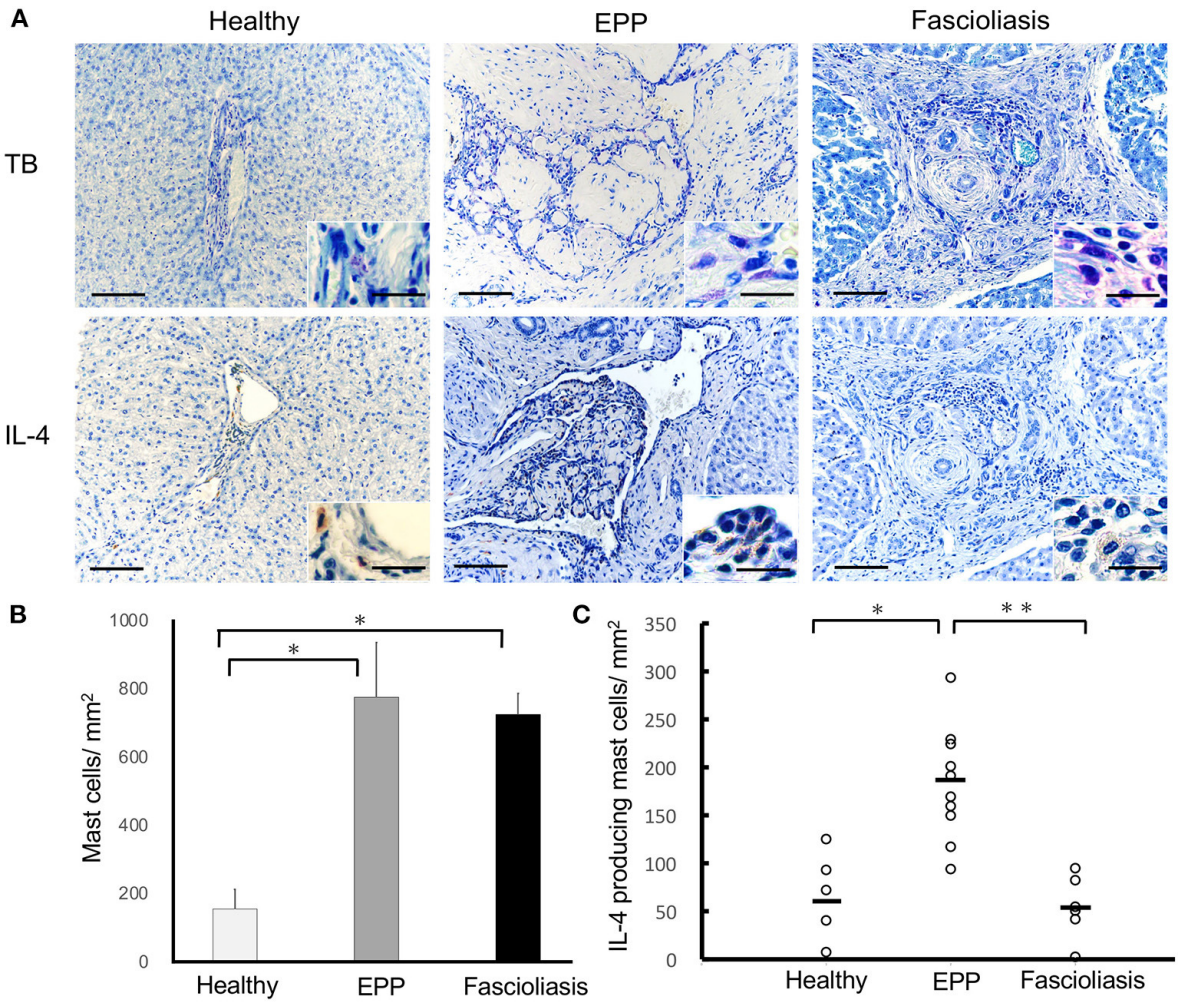
Mast cell infiltration and IL-4 production in bovine liver. **(A)** Hepatic interlobular triad region of a healthy liver (Healthy), an eosinophilic proliferative phlebitis (EPP) case, and a chronic fascioliasis case (Fascioliasis) were indicated using toluidine blue staining or immunostaining by anti-IL-4 Abs (IL-4) in hematoxylin stain background. Inset is a high-magnification image. Each scale bar indicates 50 μm in low-magnification images and 10 μm in high-magnification images. **(B)** Average numbers of mast cells per 1 mm^2^ were indicated in the healthy cattle (*n* = 5), EPP cattle (*n* = 10), and fascioliasis cattle (*n* = 6). *P < 0.02, analysis of variance (ANOVA). **(C)** Numbers of IL-4–positive mast cells per 1 mm^2^ were indicated in the healthy cattle (*n* = 5), EPP cattle (*n* = 10), and fascioliasis cattle (*n* = 6). Black bars, mean value of each cattle. *P < 0.05, ANOVA; **P < 0.04, ANOVA. IL-4, interleukin-4.

In EPP cattle, although the total number of MCs detected with metachromatic granules *via* toluidine blue staining were observed to be similar to the amount of infiltration into hepatic lesions of fascioliasis cattle, the number of MCs per 1 mm^2^ in EPP cattle (747.3 ± 87.3; *n* = *10, P* = 0.0112, one-way ANOVA, two-tailed test) or fascioliasis cattle (713.4 ± 57.6; *n* = *6, P* = 0.0163, one-way ANOVA, two-tailed test) increased significantly compared with that in healthy cattle (177.1 ± 37.4, *n* = *5*) (Figures 2A, B). However, the number of IL-4–producing MCs per 1 mm^2^ of EPP cattle (182.5 ± 75.2, *n*=*10*) was significantly higher than that in fascioliasis cattle (58.2 ± 21.2; *n* = *6, P* = 0.0392, one-way ANOVA, two-tailed test) or healthy cattle (63.1 ± 37.5; *n* = *5, P* = 0.0461, one-way ANOVA, two-tailed test) (Figures 2A, C). Thus, the ratio of IL-4–producing MCs in counted MCs infiltration in EPP cattle (24.4%) was 3 times more than in fascioliasis cattle (8.1%).

### 3.3. Role of MC tryptase/PAR-2-pathway in EPP

**Figure 3:**
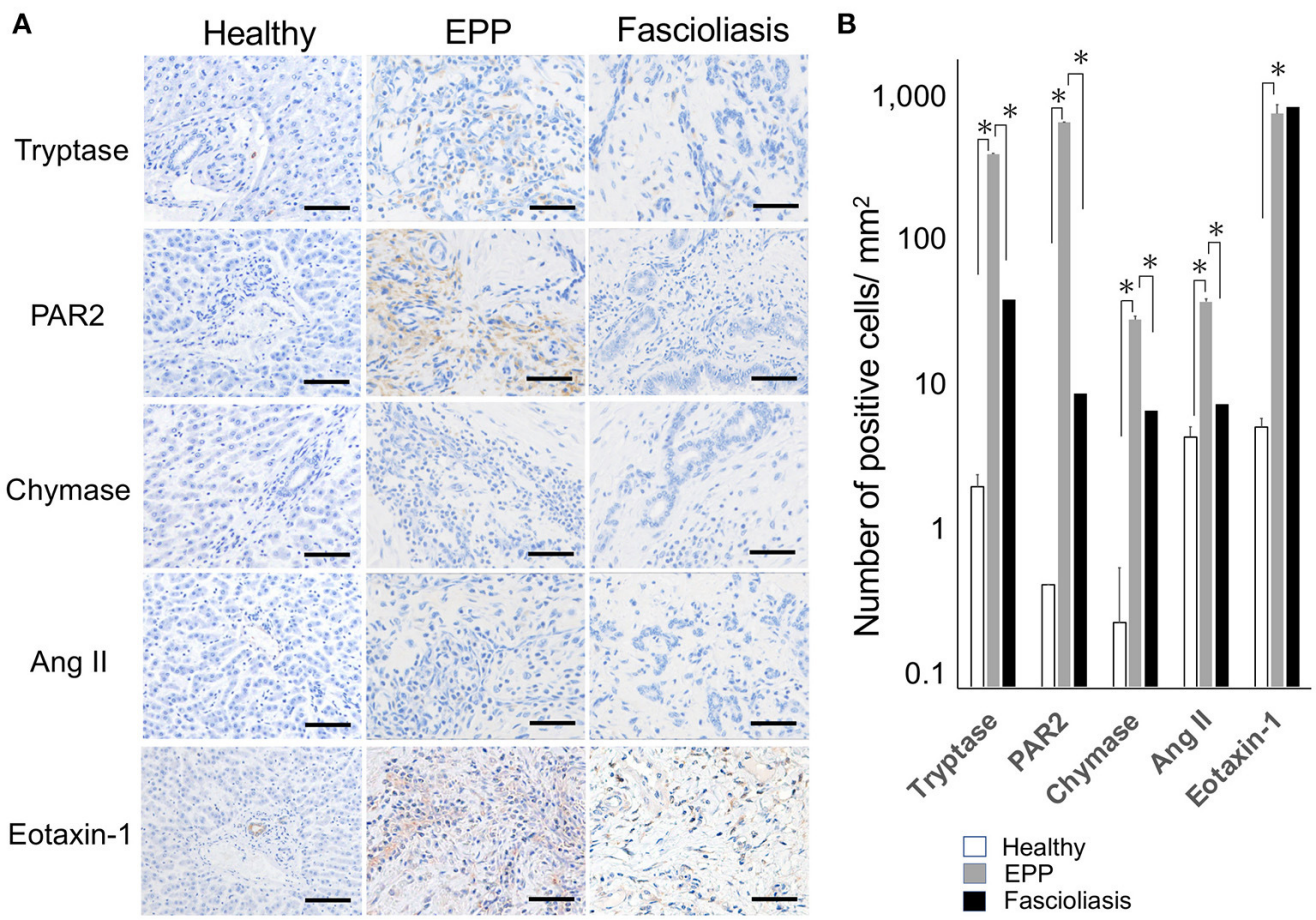
Type of mast cells containing cytoplasmic granules (tryptase and chymase) and the expression response of related molecules (PAR-2, Ang II, and eotaxine-1) in surrounding cells. **(A)** Hepatic interlobular triad region of a healthy case (Healthy), an eosinophilic proliferative phlebitis (EPP) case, and a chronic fascioliasis case (Fascioliasis) were indicated by immunostaining against tryptase, PAR-2, chymase, Ang II, and eotaxine-1. Each scale bar indicates 50 μm. **(B)** Average numbers of cytoplasmic granules (tryptase and chymase) per 1 mm^2^ and related molecules (PAR-2, Ang II, and eotaxine-1) in surrounding cells per 1 mm^2^ were indicated in healthy cattle (*n* = 5), EPP cattle (*n* = 10) and fascioliasis cattle (*n* = 6). *P < 0.05, analysis of variance. PAR-2, protease activated receptor-2; Ang II, angiotensin II.

MCs are reported to have cytoplasmic granules containing mediators, such as heparin, and enzymes, including chymase and carboxypeptitase-A, which can be found upon cell activation. MC subsets expressing both tryptase and chymase (MC^TC^) tend to be abundant in the dermis. In contrast, MC subsets expressing mainly tryptase (MC^T^) may be found in the mucosa of organ systems. A third and minor population of MCs expresses chymase and cathepsin G (MC^C^) (10). The number of MC^T^ per 1 mm^2^ in EPP cattle (267.4 ± 34.8, *n* = 10, *P* = 0.00134, one-way ANOVA, two-tailed test) increased significantly compared with that in fascioliasis cattle (19.2 ± 5.4, *n* = 6). Thus, MC^T^ ratio in MC infiltration in EPP cattle is 35.8% compared with 2.7% in fascioliasis cattle. In this context, the numbers per 1 mm^2^ of periendothelium fibroblasts expressing PAR-2 but not angiotensin II (Ang II) increased significantly in EPP cattle (626.1 ± 56.3, *n* = 10, *P* = 0.0000101, one-way ANOVA, two-tailed test) compared with that in fascioliasis cattle (1.7 ± 0.7, *n* = 6) (Figures 3A, B). Eotaxin-1–producing cells in EPP cattle (785.3 ± 71.4, *n* = 10) were detected in as many endothelium cells and fibroblasts as fibroblasts of fascioliasis cattle (802.5 ± 89.1, *n* = 6) (Figures 3A, B). In healthy cattle, the expression of tryptase, PAR-2, chymase, Ang II, and eotaxin-1 are hardly detected, but it was a little detected some mast cells expressed tryptase, some interlobular veins expressed Ang II, and some interlobular bile ducts expressed eotaxin-1.

### 3.4. Severe hypo-vitamin A and normal range of hepatic enzymes in EPP

Serum VA concentration was a little low even in healthy cattle (27-33 months age, 62.6 ± 4.8 IU/dl, *n* = 5) than the normal range 93.3 ± 14.8 IU/dl (11, 12). Furthermore, EPP cattle (25–35 months age, 30.9 ± 10.2 IU/dl, *n* = 10) were suggested to a correlation with severe hypo-VA. In fascioliasis cattle (98–169 months age), 7–12 years had passed since the fattening period of low VA, so those VA values (176.0 ± 35.7 IU/dl, *n* = 6) were significantly high than the normal range. Although serum AST (155.8 ± 35.4 IU/l, *n* = 6) and γ-GTP (111.2 ± 26.5 IU/l, *n* = 6) increase significantly in fascioliasis cattle, in EPP cattle, serum AST (89.2 ± 10.7 IU/l) and serum γ-GTP (37.2 ± 9.7 IU/l) was in the normal range (13), but those values were a little higher than in healthy cattle (70.3 ± 4.4 IU/l, 21.4 ± 6.2 IU/l, respectively) (Table 1).

**Table 1:** Serum vitamin A (retinol), AST, γ-GTP in healthy, eosinophilic proliferative phlebitis (EPP), and fascioliasis cattle.

	**Healthy**	**EPP**	**Fascioliasis**
	**(*n* = 5)**	**(*n* = 10)**	**(*n* = 6)**
Age (months)	27–33	25–35	98–169
Vitamin A (IU/dl)	62.6 ± 4.8	30.9 ± 10.2^*^	176.0 ± 35.7^**^
AST (IU/l)	70.3 ± 4.4	89.2 ± 10.7^*^	155.8 ± 35.4^**^
γ-GTP (IU/l)	21.4 ± 6.2	37.2 ± 9.7^*^	111.2 ± 26.5^**^

Data indicate average ± standard deviation. Statistical differences to the effect of healthy cases were detected in EPP cases. ^*^P < 0.05, analysis of variance (ANOVA); Statistical differences to the effect of healthy and EPP cases were detected in fascioliasis cases. ^**^P < 0.05, ANOVA. EPP, eosinophilic proliferative phlebitis.

## 4. Discussion

This study indicated that type 2 helper T cell (Th2) response with IL-4–producing MC^T^ type (high tryptase/PAR-2, low chymase/Ang II) significantly dominates in EPP cattle than in fascioliasis cattle (Figure 3), that is, suggesting that EPP cattle will be triggered by type I hypersensitivity (allergic disease) (10). Because it was reported that MC tryptase/PAR-2 activation participates in liver injury through the activation of liver sinusoidal endothelial cells (14), local circulatory disorders in liver may have occurred in EPP cattle. Although it was believed that Th2 responses are strongly induced on immune responses of bovine fascioliasis, IgG1 and IgE reactions in Th2 responses differ immunologically (15). In humans and mice, type 1 helper T cell (Th1) and Th2 regulate the development of the opposite IgG subsets reciprocally in plasma cells by secreting interferon-γ (IFN-γ) and IL-4 (16). In cattle, IgG subclasses are regulated with Th1/Th2 cytokines like those in mice rather than in humans; that is, IL-4 promotes IgG1 and IFN-γ promotes IgG2 (16). To date, several previous reports have suggested an early and predominant response of IgG1 with a simultaneous expression of delayed and weak IgG2 in experimental *F. hepatica*- or *F. gigantica-*infected cattle (17, 18). Although kinetics of the serum immunoglobulin isotype response in bovine fascioliasis exhibited high IgG1 and weak IgG2 and IgA after early IgM, there is no evidence pertaining to type I allergic response with IL-4–positive MC activation through IgE (18). Recently, the immune mechanism in bovine fascioliasis was indicated to be inhibited *via* regulatory T cell (Treg) (19, 20), including some key immunological pathways of natural killer (NK) cell activity and IgE-mediated signaling (21). In fascioliasis cattle, unique MCs producing neither tryptase nor chymase infiltrated, and no expression of PAR-2 and angiotensin II was observed in surrounding cells (Figure 3). The infiltration of unique MC types in fascioliasis cattle is thought to indicate MC activation through not IgE but epithelial cell cytokines IL-33 and so on (9, 10).

Some recent studies revealed differences between adaptive immunity and innate immunity, i.e., IL-4 production of MCs crosslinked IgE specific to allergens and a parasite response *via* ILC2 (9, 10). ILC2 cells do not express antigen-specific receptors and are activated by epithelial cell cytokines IL-25, IL-33, and thymic stromal lymphopoietin (9, 10). In bovine fascioliasis, Th2 responses with suppression *via* Treg could be related to IgG1, not IgE, in the class switch of plasma cells (19–21). To the best of our knowledge, this is the first report that distinguishes allergic lesions from fascioliasis lesions by detecting remarkable infiltration of IL-4– and tryptase-producing MC^T^s in the periendothelium *via* immunohistochemistry (Figures 2, 3). Overall, this study supports that EPP cases indicate not bovine fascioliasis but chronic hypersensitivity. These processes were summarized in Supplementary Figure 1.

EPP cases suggested to a correlation with severe serum hypo-vitamin A (Table 1). The frequent occurrence of EPP cattle in Japan may be able to explained as follows. In beef production, beef quality and beef quantity are two of the most critical factors in the beef grading system in Japan. In Japan, beef on an adaptive degree of “marbled meat” with fatty infiltration is traded at an inflated price that is 1.5–2.0 higher than “reddish meat.” VA was first found to be negatively correlated with marbling score in carcasses of Japanese Black steers (22). This was supported by numerous studies on adipocyte development with VA restriction strategy in beef cattle during fattening period (23). To produce effectively marbled meat, many Japanese farmers feed cattle with forage containing a lower level of VA during the fattening stage of calves. The prevalence of EPP has increased throughout Japan since late 1990s when VA-deficient feed was recommended by many prefectural governors (1). VA deficiency has been reported to increase sensitivity to allergy with a Th2 shift in JB cattle (24) or laboratory mice (25). VA deficiency has been reported to increase the risk of Treg-to-Type 17 helper T cell (Th17) reprogramming, resulting in the induction of enteritis (26) and IgE-mediated food allergy (27); that is, it was suggested to enhance allergic responses *via* Th17 because Treg with the immunosuppressive function is reduced by VA deficiency. Although we previously reported the enteritis with severe eosinophilic infiltration in EPP cattle (3), a recent case report in Japan on eosinophilic enteritis with diarrhea in JB fattening cattle (28) may be linked to the process of lesion formation of EPP cases.

EPP cattle were indicated to have not occurred liver injury with raised hepatic enzymes (AST, γ-GTP) like bovine fascioliasis (Table 1). If EPP cases had no also clinical signs, that will be normally shipped as healthy. That is, it is not until being slaughtered that EPP lesion is detected in a cow shipped as healthy. This study showed that EPP cases sometimes occurring in Japan may be an allergic disease brought by social economic circumstances, and not the epidemics of bovine fascioliasis. We believe that the academically clarification on EPP cases is important for food safety.

## Data availability statement

The raw data supporting the conclusions of this article will be made available by the authors, without undue reservation.

## Ethics statement

Ethical review and approval was not required for the animal study because for research using the liver of cattle discarded after meat inspection.

## Author contributions

KO-T and MK contributed to conception and design of the study. KO-T organized the database. MK and KT performed the statistical analysis. MK wrote the first draft of the manuscript. KO-T, YM, and MM wrote sections of the manuscript. All authors contributed to manuscript revision, read, and approved the submitted version.
