# Supplementary material for: Intrahepatic eosinophilic proliferative phlebitis in Japanese black cattle indicate allergies involving mast cell tryptase-dependent activation
Source: Front Vet Sci. 2022 Dec 20;9:972180. doi: 10.3389/fvets.2022.972180 (PMC9807620; doi:10.3389/fvets.2022.972180)
Supplement: Supplementary file 1 [file Data_Sheet_1.PDF]

Supplementary Material

1.1 Supplementary Figures

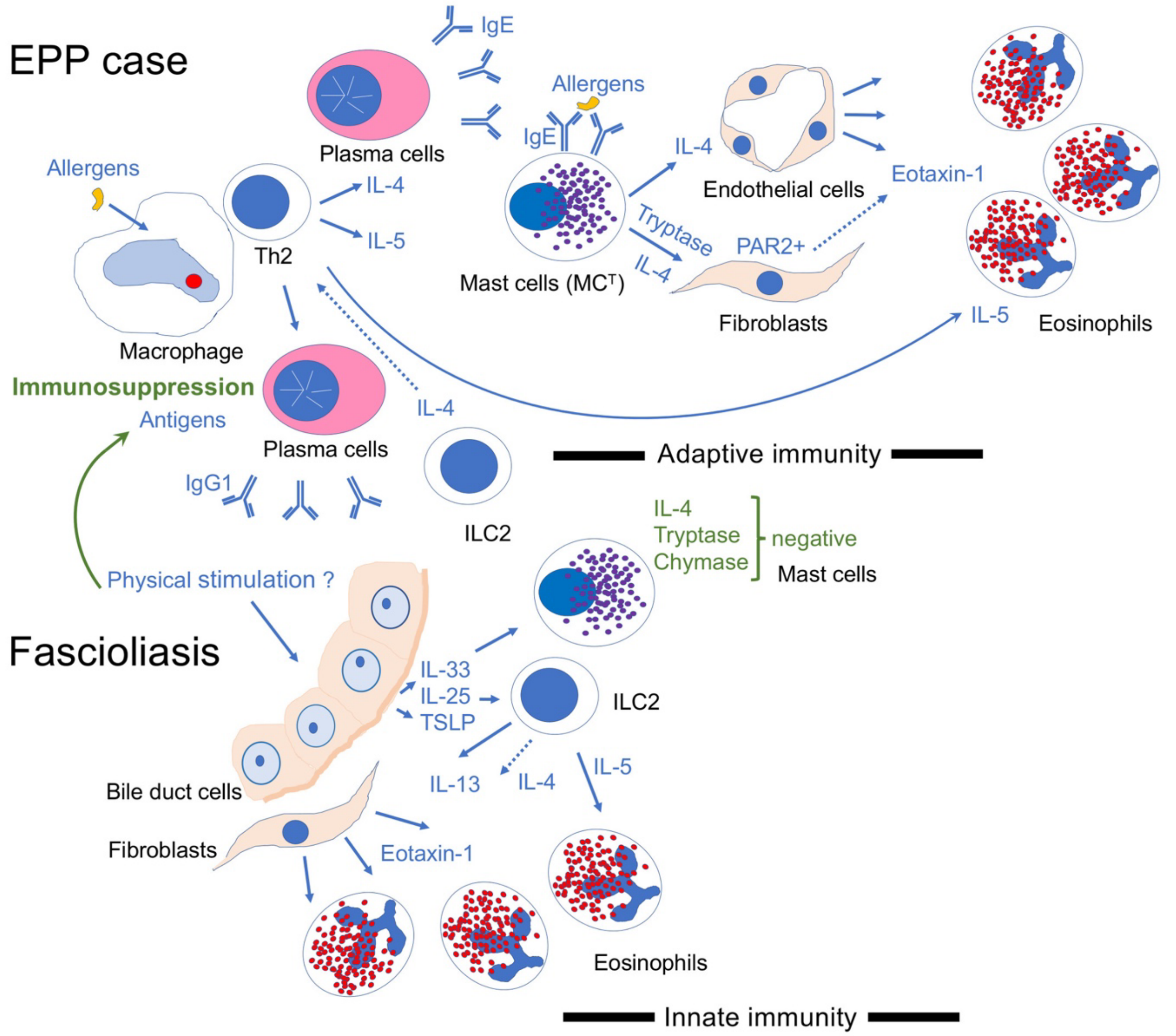

Supplementary Figure 1. Differences in the mechanism of eosinophilic infiltration via adaptive immunity and innate immunity.

In fascioliasis, biliary epithelium, which is stimulated by the *Fasciola* spp. parasite in the bile duct, produces IL-33, IL-25, and TSLP. IL-33 activates MCs by IgE-independent, and IL-33, IL-25, and TSLP induce ILC2. Then, fibroblasts activated by ILC2 induce eosinophilic infiltration via eotaxin-1 production. In eosinophilic proliferative phlebitis (EPP) cases, MC^T crosslinked by IgE specific to allergen produces IL-4. Endothelium cells and PAR2-expressing periendothelium fibroblasts respond to IL-4 induce eosinophilic infiltration via eotaxin-1. IL, interleukin; TSLP, thymic stromal lymphopoietin; MC, mast cell; MC^T, MC subset expressing mainly tryptase; IgE, immunoglobulin E; ILC2, group-2 innate lymphoid cell; PAR-2, protease activated receptor-2
